# Predicting malignancy in thyroid nodules: feasibility of a predictive model integrating clinical, biochemical, and ultrasound characteristics

**DOI:** 10.1186/s13044-016-0033-y

**Published:** 2016-05-25

**Authors:** Justyna Witczak, Peter Taylor, Jason Chai, Bethan Amphlett, Jean-Marc Soukias, Gautam Das, Brian P. Tennant, John Geen, Onyebuchi E. Okosieme

**Affiliations:** Centre for Endocrine and Diabetes Science, Institute of Molecular and Experimental Medicine, Cardiff University School of Medicine, University Hospital Wales, Cardiff, CF14 4XN UK; Radiology Department, Prince Charles Hospital, Cwm Taf Health Board, Gurnos Estate Merthyr Tydfil, CF47 9DT UK; Clinical Biochemistry Department, Prince Charles Hospital, Cwm Taf Health Board, Gurnos Estate Merthyr Tydfil, CF47 9DT UK; Endocrinology and Diabetes Department, Prince Charles Hospital, Cwm Taf Health Board, Gurnos Estate Merthyr Tydfil, CF47 9DT UK; Faculty of Life Sciences and Education, University of South Wales, Glyntaff Campus, Pontypridd, CF37 1DL UK

**Keywords:** Thyroid cancer, Thyroid nodule, Diagnosis, Predictive model, TSH, Ultrasound

## Abstract

**Background:**

Although the majority of thyroid nodules are benign the process of excluding malignancy is challenging and sometimes involves unnecessary surgical procedures. We explored the development of a predictive model for malignancy in thyroid nodules by integrating a combination of simple demographic, biochemical, and ultrasound characteristics.

**Methods:**

Retrospective case-record review.

We reviewed records of patients with thyroid nodules referred to our institution from 2004 to 2011 (*n* = 536; female 84 %, mean age 51 years). All malignancy was proven histologically while benign disease was either confirmed histologically, or on cytology with minimum 36-month observation period. We focused on the following predictors: age, sex, smoking status, thyroid hormones (FT4 and TSH) and nodule characteristics on ultrasound. Variables were included in a multivariate logistic regression and bootstrap analyses were used to confirm results.

**Results:**

Independent predictors of malignancy in the fully adjusted model were TSH (OR 1.53, 95 % CI 1.10, 2.12, *p* = 0.01), male gender (OR 3.45, 95 % CI 1.33, 8.92, *p* = 0.01), microcalcifications (OR 6.32, 95 % CI 2.82, 14.1, *p* < 0.001), and irregular nodule margins (OR 5.45, 95 % CI 1.61, 18.6, *p* = 0.006) Bootstrap analyses strengthened these associations and a parsimonious analysis consisting of these variables and age-group demonstrated an area under the curve of 0.77. A predictive score was sensitive (86.9 %) at low scores and highly specific (94.87 %) at higher scores for distinguishing benign from malignant disease.

**Conclusions:**

A predictive model for malignancy using a combination of clinical, biochemical, and radiological characteristics may support clinicians in reducing unnecessary invasive procedures in patients with thyroid nodules.

**Electronic supplementary material:**

The online version of this article (doi:10.1186/s13044-016-0033-y) contains supplementary material, which is available to authorized users.

## Background

Thyroid cancer is the most common endocrine malignancy accounting for more deaths than all other endocrine cancers combined [1]. The incidence of thyroid cancer has continued to increase worldwide, partly due to greater diagnostic activity, as well as a true increase in disease occurrence [2, 3]. The differentiated thyroid cancers, papillary and follicular, are the most prevalent histological types with papillary cancer accounting for 40–70 % of all cases of thyroid cancer [1]. In recent years small papillary microcarcinomas, less than one cm in size, are now increasingly detected due to the growing use of modern imaging techniques such as ultrasound, computerised tomography or positron emission tomography (PET) scans [4]. Although differentiated thyroid cancers generally carry a favourable prognosis affected individuals suffer considerable morbidity, both from the disease as well as from side effects of therapy. In most instances treatment with thyroidectomy and radioactive iodine ablation will incur the need for lifelong thyroid hormone therapy [5].

Patients with thyroid cancer commonly present with clinically or radiologically detectable thyroid nodules [6, 7]. The prevalence of palpable thyroid nodules is estimated at about 4-7 % of the adult population in iodine-sufficient areas [6] with higher figures reported in women, older patients, and in studies based on ultrasound scan examination [6–8]. Yet despite the high frequency of thyroid nodules only about 5–10 % of such nodules are malignant [6, 7]. Fine needle aspiration biopsy (FNAB) and histology is the gold standard for diagnosing thyroid cancer but up to 25 % of biopsies performed in practice are non-diagnostic [9]. Patients with non-diagnostic biopsies often undergo repeat biopsies and in some cases proceed to a diagnostic thyroidectomy with its accompanying risks of permanent hypoparathyroidism and recurrent laryngeal nerve damage [10].

The challenge for the clinician is thus to predict which thyroid nodules are malignant as patients with benign nodules can be spared unnecessary intervention. A number of simple clinical characteristics have traditionally been associated with an increased malignancy risk including male gender, age < 30 years or >60 years, a family history of thyroid cancer, previous exposure to head and neck irradiation, and a firm, hard, rapidly enlarging nodule with compressive symptoms such as dysphagia, dysphonia and breathlessness [6]. Ultrasound examination of the thyroid is also a useful adjunct to clinical assessment, and nodule characteristics that carry a high risk of malignancy include hypoechogenicity, microcalcifications, irregular margins, solid composition, and single nodularity [11]. However when considered individually none of these features are sufficiently sensitive to guide clinical decisions [12].

In recent years serum thyrotropin (TSH) has emerged as an independent predictor of malignancy in patients with thyroid nodules [13, 14]. TSH is a thyroid growth factor and in animal experiments stimulates thyroid cell proliferation through cyclic AMP mediated pathways, thereby promoting thyroid malignancy [15]. Furthermore, levothyroxine-induced TSH suppression remains a therapeutic cornerstone for preventing cancer recurrence following thyroidectomy and radioactive iodine treatment [16]. In observational studies a direct relationship has been shown between TSH concentrations and thyroid cancer risk and other adverse outcomes even at TSH levels within the euthyroid range [17, 18]. In addition increased TSH concentration is associated with a more advanced stage of thyroid cancer at presentation [19] adding credence to its pivotal role in the development and progression of thyroid cancer.

Thus, the prediction of thyroid cancer in patients with thyroid nodules involves multiple clinical, biochemical, and radiological risk factors and a predictive model is unlikely to be satisfactorily achieved by a single set of predictors. While most studies to date have focused on single risk factors, whether clinical, radiological, or biochemical, only a few studies have evaluated these factors in combination. A robust predictive model incorporating a combination of simple easily obtainable clinical, laboratory and radiological risk factors may offer adequate sensitivity to serve as a pragmatic decision aid to support clinicians in practice. The aim of this study therefore was to explore the potential of developing a predictive model for the risk of thyroid cancer in patients with thyroid nodules using demographic, clinical, laboratory and ultrasound predictors.

## Methods

### Patients

We retrospectively reviewed the records of patients referred for evaluation of thyroid nodules at our institution between January 2004 and December 2011. Patients with thyroid nodules were identified from histopathology records (*n* = 602). We excluded patients with inadequate clinical or laboratory information and patients in whom a final diagnosis was not reached (*n* = 66). All diagnoses of malignancy were proven histologically after thyroidectomy or surgical biopsy (*n* = 50). Benign disease was either confirmed histologically in patients following thyroidectomy or surgical biopsy (*n* = 362) or was based on a negative fine needle aspiration cytology and a minimum clinical observation period of 36 months in those who did not undergo surgery (n = 124). We evaluated the following candidate predictors: age, gender, smoking status, thyroid hormone status i.e. serum free thyroxine (FT4) and serum TSH, and thyroid ultrasound characteristics (hypoechogenicity, microcalcifications, irregular margins, spherical shape, and single nodularity and solid composition). Approval to undertake the study was received from the NHS Research and Ethics Committee for Wales (reference 12/WA/0334) and the Cwm Taf University Health Board Research and Development Office (reference CT/278/101463/12).

### Statistical analysis

To select suitable predictors in the model all *apriori* candidate predictors were entered initially into a univariate analysis and then all variables were included in a multivariate logistic regression to analyse the odds of developing malignancy. The relevant factors and their weighting were then built into a final model. TSH and FT4 levels were categorized into tertiles. As the risk of malignancy is increased in young adults as well as the elderly we coded age as 1 if the age was less than 30 years or more than 60 years while patients aged 30–60 years were coded as 0. Smokers were defined as never smoked, previous smokers, or current smokers. Ultrasound characteristics such as size, echogenicity and nodule composition were utilised as ordered categorical variables. Logistic regression was undertaken to assess the odds of malignancy for each of these variables. In addition we explored for interaction between TSH level and age-group and TSH and gender using likelihood ratio tests. We then modelled in a parsimonious approach all variables associated with malignancy in univariate or multivariate analysis maintaining age-group and gender as forced variables. Confirmatory bootstrap analysis was undertaken with 1000 iterations using age-group and gender as strata. These variables were taken forward for area under the receiver operating characteristic (ROC) using the “lroc” command in STATA. A risk score was then generated based on identified associations and the magnitude of their effect. All statistical analysis was undertaken using STATA version 12 (STATACORP, College Station, TX, USA).

## Results

### Baseline characteristics

We identified 602 patients from the thyroid histopathological records. Of these, 48 (8 %) did not have a final cytological or histological diagnosis and clinical records were irretrievable in 18 (3 %) patients leaving a final sample size of 536 for analysis. Data on smoking was missing for 81 individuals while 22 individuals had missing data for TSH. The baseline characteristics of these patients is summarised in Table 1. As expected the majority of our cohort was female, young to middle age, with a 9 % malignancy detection rate. The histological subtypes comprised papillary (69 %), follicular (8 %), hurtle cell (4 %), anaplastic (6 %), lymphoma (6 %), and medullary cancer (6 %). Of the 536 patients 70 (13 %) had indeterminate cytology including atypical cytology (Thy3a) and suspected follicular neoplasm (Thy3f) [5]; of these 5 patients (7 %) had a final diagnosis of malignancy.

**Table 1:** Baseline characteristics of patients

Characteristics	Benign	Malignant	Total
Number	486	50	536 (100 %)
Male sex (%)	67 (13.8 %)	19 (38.0 %)	86 (16.0 %)
Age (years)	51.2 (15.9)	50.9 (15.9)	51.2 (15.9)
Smoking status			
Never	240 (57.4 %)	23 (62.1 %)	263 (57.8 %)
Previous	53 (12.7 %)	3 (8.11 %)	56 (12.3 %)
Current	125 (29.9 %)	11 (29.7 %)	136 (29.9 %)
Thyroid status			
TSH (mU/l)	1.20 (0.50–2.00)	1.95 (1.13–2.90)	1.40 (0.70–2.40)
FT4 (pmol/l)	15.8 (7.52)	13.8 (5.38)	16.9 (7.56)
Nodule size (cm)	2.91 (1.63)	2.99 (1.44)	2.91 (1.61)
Microcalcification	61 (12.5 %)	26 (52.0 %)	87 (16.2 %)
Nodule Composition			
Cystic	90 (18.5 %)	6 (12.0 %)	96 (17.9 %)
Mixed	53 (10.9 %)	6 (12.0 %)	59 (11.0 %)
Solid	343 (70.6 %)	38 (76.0 %)	381 (71.1 %)
Echogenicity			
Iso or hyper-echoic	332 (68.3 %)	25 (50.0 %)	357 (66.6 %)
Mixed	59 (12.1 %)	9 (18.0 %)	68 (12.7 %)
Hypo-echoic	95 (19.6 %)	16 (32.0 %)	111 (20.7 %)
Margins			
Regular	469 (96.5 %)	43 (86.0 %)	512 (95.5 %)
Irregular	17 (3.50 %)	7 (14.0 %)	24 (4.5 %)

Figures are mean (SD), number (%) or median (IQR)

### Predictors of malignancy

Independent predictors of malignancy in the fully adjusted model were TSH group (OR 1.53, 95 % CI 1.10, 2.12, *p* = 0.01), male gender (OR 3.45, 95 % CI 1.33, 8.92, *p* = 0.01), the presence of microcalcifications (OR 6.32, 95 % CI 2.82, 14.1, *p* < 0.001), and irregular margins (OR 5.45, 95 % CI 1.61, 18.6, *p* = 0.006) (Table 2). The association between TSH levels and malignancy was only marginally attenuated when adjusting for FT4. In this relationship, we found no evidence of interaction between TSH levels and age-group (*p* = 0.91) or gender (*p* = 0.64). Bootstrap analysis confirmed the key associations with all associations remaining statistically significant (Table 3). A parsimonious analysis consisting variables associated with malignancy and forced variables demonstrated an area under the curve of 0.77 (Additional file 1: Figure S1).

**Table 2:** Odds Of Malignancy In Univariate And Multivariate Analyses

	Univariate analysis (*n* = 536)		Multivariate analysis (*n* = 422)	
Variable	OR (95%CI)	p^a^	OR (95%CI)	p^a^
TSH – upper tertile	1.52 (1.19, 1.92)	0.001	1.53 (1.10, 2.12)	0.01
FT4 - upper tertile	0.82 (0.66, 1.02)	0.08	0.84 (0.61, 1.15)	0.28
Age-group (<30 or > 60 yrs)	1.16 (0.64, 2.09)	0.62	1.17 (0.51, 2.71)	0.71
Male gender	3.83 (2.04, 7.17)	<0.001	3.45 (1.33, 8.92)	0.01
Smoker	0.93 (0.64, 1.38)	0.75	1.02 (0.65, 1.60)	0.93
Size group	0.79 (0.55, 1.15)	0.23	0.78 (0.45, 1.37)	0.40
Microcalcification	7.55 (4.07, 14.0)	<0.001	6.32 (2.82, 14.1)	<0.001
Nodularity	1.09 (0.59, 2.01)	0.78	0.79 (0.36, 1.76)	0.57
Hypo-echogenicity	1.52 (1.09, 2.10)	0.01	1.17 (0.73, 1.89)	0.51
Irregular margins	4.49 (1.76, 11.4)	0.002	5.45 (1.61, 18.6)	0.006
Solid composition	1.24 (0.82, 1.87)	0.31	1.05 (0.62, 1.77)	0.84

^a^Calculated using the Wald Test; Multivariate analysis is analysis adjusted for all variables shown in this table

**Table 3:** Odds of malignancy using a parsimonius approach with confirmatory bootstrap analysis

	Multivariate Analysis (*N* = 514)		Bootstrap analysis (*N* = 514)	
Variable	Odds Ratio (95%CI)	p^a^	Odds Ratio (95%CI)	p^a^
TSH – upper tertile	1.65 (1.28, 2.14)	<0.001	1.65 (1.28, 2.13)	<0.001
Age-group <30 or >60 years	1.20 (0.60, 2.37)	0.61	1.20 (0.57, 2.47)	0.63
Male gender	4.41 (2.06, 9.46)	<0.001	4.41 (1.97. 9.89)	<0.001
Microcalcification	7.33 (3.62, 14.8)	<0.001	7.33 (3.35, 16.1)	<0.001
Echogenicity	1.24 (0.82, 1.86)	0.30	1.24 (0.80, 1.91)	0.34
Irregular margins	3.18 (1.05, 9.63)	0.04	3.18 (0.95, 10.6)	0.06

^a^Calculated using the Wald Test, Multivariate analysis is analysis adjusted for all variables shown in this table

### Score generation

Based on our above analysis we generated a score to predict malignancy. We used TSH > 2.5 = 1, Age-group <30 or >60 = 1, presence of microcalcification = 2, male gender = 2, irregular nodule margins = 2, mixed echogenicity = 1, and hypoechogenicity = 2. Overall, scores >4 were highly sensitive (86.9 %) whereas >7 were highly specific (94.87 %) for malignancy (Table 4). Out of 70 patients with indeterminate cytology, 40 patients (57 %) had a score of 2 or less of which 2 patients (5 %) had a final diagnosis of malignancy while 21 patients (30 %) with a score of 1 or less all had a final diagnosis of benign nodules. Thus in patients with indeterminate cytology, the negative predictive value of a score of 2 or less was 95–100 %.

**Table 4:** Sensitivity, specificity and correct classifaction of malignancy based on predictive score

Cut-point	Sensitivity	Specificity	Correctly Classified	LR+	LR-
(> = 2)	100 %	0.00 %	8.95 %	1.00	
(> = 3)	93.48 %	28.21 %	34.05 %	1.30	0.23
(> = 4)	86.96 %	52.78 %	55.84 %	1.84	0.25
(> = 5)	65.22 %	72.65 %	71.98 %	2.38	0.48
(> = 6)	45.65 %	87.82 %	84.05 %	3.75	0.62
(> = 7)	28.26 %	94.87 %	88.91 %	5.51	0.76
(> = 8)	23.91 %	97.86 %	91.25 %	11.19	0.78
(> = 9)	13.04 %	99.15 %	91.44 %	15.26	0.88
(> = 10)	4.35 %	99.79 %	91.25 %	20.35	0.96

The positive likelihood ratio (LR+) is the ratio of the probability of a positive test among the truly positive subjects to the probability of a positive test among the truly negative subjects. The negative likelihood ratio (LR–) is the ratio of the probability of a negative test among the truly positive subjects to the probability of a negative test among the truly negative subjects

## Discussion

As in other countries we have previously reported an increase in the incidence of thyroid cancer in Wales, a trend which appears predominantly driven by the growing detection of papillary thyroid cancers [20]. In parallel we have also seen a steady increase in the number of thyroid surgical procedures performed for malignant thyroid disease in the same population [21]. The patient with a thyroid nodule thus presents a challenge as it is crucial to accurately identify the small proportion of malignant cases and at the same time avoid subjecting individuals with benign nodules to unwarranted surgical interventions with lifelong repercussions. A robust predictive model for malignancy could therefore support clinicians and patients in arriving at an informed decision regarding the urgency and scope of intervention especially where FNAB is considered inconclusive or inadequate for analysis.

Our multivariate analysis confirms some well-established risk factors for thyroid malignancy including male gender, TSH > 2.5 mU/L, and typical ultrasound characteristics of microcalcification, irregular nodule margin, and solid nodule composition [22]. However, we found no association between age (<30 or >60 years) and risk of malignancy. The reason for this is unclear but may indicate a changing epidemiology with increasing detection of thyroid cancer across all ages as shown in recent surveys from our population and others [20, 23]. Thus the majority of the power for prediction was derived from the thyroid ultrasound findings although the inclusion of TSH and gender further improved the model.

Using a scoring system of 2-10 we were able to stratify the risk of malignancy in patients presenting with thyroid nodules. Our score had high specificity (95 %) at high levels (>7) but only a small proportion (4 %) of our cohort fell into this category. Nonetheless our score would be particularly useful for risk stratification in individuals with inconclusive FNAB or where invasive investigations are considered unduly intrusive such as in the frail, elderly, or in pregnancy. In such scenarios higher scores would dictate an aggressive approach while lower scores would support clinicians in adopting a more conservative or “wait and watch” policy. In our sample a score of 2 or less was associated with a low probability of disease in patients with indeterminate cytology (negative predictive value 95–100 %) and thus low scoring individuals with indeterminate cytology could have been safely managed conservatively.

A number of studies have developed predictive models for thyroid cancer in patients with thyroid nodules using a variety of approaches. Stojadinovic *et al* used Bayesian analysis to build a clinical decision aid based on a prospective analysis of 216 patients with thyroid nodules who underwent thyroidectomy [24]. Their model incorporated nodule size, FNAB cytology, and ultrasound characteristics, and was highly predictive of malignancy (AUC 0.88, 95 % CI; 0.82-0.94). However this model was also based on the technique of electrical impedance scanning which is not routinely available in practice [24]. Other studies utilising similar Bayesian principles have reported various combinations of clinical and sonographic predictors such as age, gender, and ultrasound characteristics [25, 26]. One study utilised a classification and regression tree (CART) classifier in a two-stage model (sensitivity 80 %, specificity 94.1 %) that included age, sex, and nodule size in the first stage and echogenicity and microcalcifications in the second stage [27].

Another approach used by others has been to develop risk stratification algorithms derived from multivariate analysis. Boelaert and co-workers calculated a predictive formula for malignancy in patients with nodules, using age, gender, goitre type, and serum TSH [14]. In their study a TSH in the upper third of the normal range was significantly associated with malignancy (OR 2.91 95 % CI 1.49, 5.71) [14]. By means of a regression model Maia et al showed a strong risk of malignancy in patients aged >39 years and in those with suspicious ultrasound features (predictive accuracy, 81.7 %) [28]. Similar findings were reported in another study restricted to individuals with indeterminate or suspicious FNABs [29]. Elsewhere, Nixon *et al* applied 8 predictor variables including TSH, ultrasound characteristics, and FNA cytology to create a nomogram with a high discriminatory ability (concordance index, 91 %) [30]. One mathematical formula derived from ultrasound characteristics alone proved reliable for detecting non-follicular neoplasms (sensitivity, 86.5 %, specificity 92.3 %) [31]. However the utility of complex calculations and algorithms in busy clinical settings is still unclear.

The strength of our study lies in the simplicity and practical appeal of our predictive score as an office based tool derived from a combination of easily obtainable predictors. As a decision aid our score could inform clinicians and potentially limit the number of unnecessary invasive procedures undertaken in low-risk individuals with inconclusive or inadequate FNABs. Furthermore our data, sourced from a relatively large clinic population, is representative of patients with thyroid nodules encountered in everyday practice. The malignancy rate of 9 % in our sample is consistent with the prevalence of malignancy in secondary care referral settings. However our study has some limitations. Although all cases of malignancy were diagnosed by surgical specimen histology some individuals with benign disease did not undergo surgery but relied on the FNAB and a three year clinical observation period for the final diagnosis. Nonetheless this approach reduced surgical selection bias allowing us to analyse a broader spectrum of patients with a greater percentage of low risk individuals. Data were not available on anti-TPO antibody status for the majority in patients therefore this was not included in models. Lastly our data was retrospective and will have to be considered preliminary pending confirmation in an externally validated prospective cohort.

## Conclusion

In conclusion a predictive model for malignancy using a combination of simple accessible clinical, biochemical, and sonographic predictors could improve the management of thyroid nodules by supporting clinicians in reducing invasive surgical procedures in low risk patients with thyroid nodules.

## Abbreviations

AMP, Adenosine monophosphate; AUC, area under the curve; CI, Confidence interval; FNAB, Fine needle aspiration biopsy; FT4, Free thyroxinel; OR, Odds ratio; PET, Positron emission tomography; ROC, receiver operating characteristics; TSH, Thyroid stimulating hormone

## Additional file

Additional file 1: Figure S1. Area under the curve for model predicting malignancy. Area under ROC curve = 0.7667 (PDF 14 kb)
