# Supplementary figures and images for: Predicting malignancy in thyroid nodules: feasibility of a predictive model integrating clinical, biochemical, and ultrasound characteristics
Source: Thyroid Res. 2016 May 25;9:4. doi: 10.1186/s13044-016-0033-y (PMC4910190; doi:10.1186/s13044-016-0033-y)

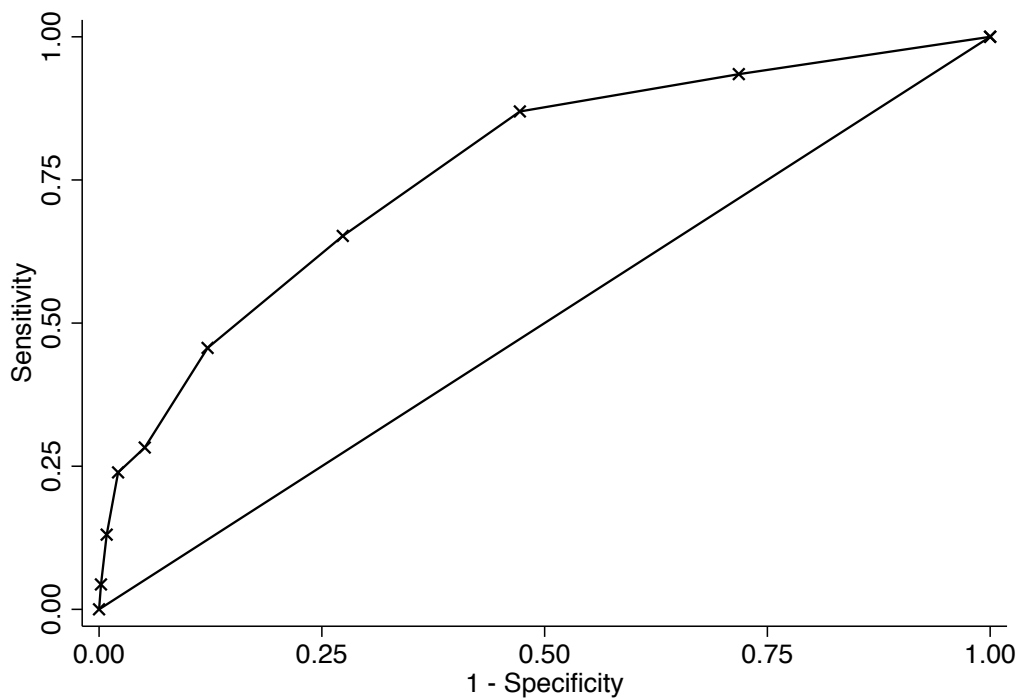

Area under ROC curve = 0.7667

Supplement: Additional file 1: Figure S1. — Area under the curve for model predicting malignancy. Area under ROC curve = 0.7667 (PDF 14 kb) [file 13044_2016_33_MOESM1_ESM.pdf]
